# International collaboration and networking in academic medicine through social media

**DOI:** 10.1192/j.eurpsy.2023.190

**Published:** 2023-07-19

**Authors:** V. Pereira-Sanchez

**Affiliations:** Child and Adolescent Psychiatry, New York University Grossman School of Medicine, New York, United States

## Abstract

**Abstract:**

Dr. Pereira-Sanchez will draw upon his experience of use of various major social media platforms for professional networking and collaboration in international psychiatry and global mental health. These platforms offer opportunities to connect with colleagues worldwide individually and in groups and open avenues for peer support, research and advocacy. Specific examples the speaker will bring from his own experience include the World Network of Psychiatric Trainees and the Global Mental Health Think Tank, both of which gather hundreds of colleagues across continents.

**Disclosure of Interest:**

None Declared

